# Hospitalizations associated with rotavirus gastroenteritis in Spain, 2001–2005

**DOI:** 10.1186/1471-2458-8-109

**Published:** 2008-04-08

**Authors:** Ana López-de-Andrés, Rodrigo Jiménez-García, Pilar Carrasco-Garrido, Alejandro Alvaro-Meca, Patricia Graciela Galarza, Ángel Gil de Miguel

**Affiliations:** 1 Preventive Medicine and Public Health Teaching and Research Unit. Health Sciences Faculty. Rey Juan Carlos University. Avda de Atenas s/n, Alcorcón 28402 Madrid, Spain

## Abstract

**Background:**

This study aims to describe and analyze hospital admissions in Spain due to rotavirus infections among children aged 5 years or under during the period 2001–2005, along with the associated health cost.

**Methods:**

To update estimates of rotavirus hospitalizations rates in Spain, we conducted a retrospective study of 5 years of national hospitalization data associated with acute gastroenteritis using the Minimum Basic Data Set.

**Results:**

During the study period, a total of 17.1% of all admissions due to acute gastroenteritis of any etiology in children aged ≤ 5 years were attributable to rotavirus infection as determined by the rotavirus-specific International Classification of Diseases, ninth revision, Clinical Modification code. A mean incidence of 135 hospital admissions attributable to rotavirus per 100,000 children aged ≤ 5 years was found. Hospitalizations associated with rotavirus had a marked winter-time seasonality. The estimated cost of hospital admission attributable to rotavirus has risen from 3 million euros estimated for 2001 to almost 7 million euros estimated in 2005.

**Conclusion:**

Rotavirus gastroenteritis remains an important cause of hospitalizations in Spanish children, mostly during the winter season.

## Background

Rotavirus is regarded as the most frequent cause of acute gastroenteritis among children under 5 years of age [1]. Indeed, in the USA, rotavirus generates 410,000 medical consultations, 205,000–272,000 emergency visits, and 55,000–70,000 hospital admissions per year [2-5]. Estimates show that in 2006, Europe had 3.6 million cases per year among children aged under 5 years, with 231 deaths, in excess of 87,000 admissions, and around 700,000 medical consultations [6]. Currently, this infection places a considerable economic burden on the health systems of developed countries, made up of the direct cost of consultations, emergency visits and hospital admissions, plus indirect costs, such as days of work lost by parents [7,8]. The overall cost of rotavirus-induced diarrhea in the USA has been calculated as being close on one billion dollars [2-5]. A recent update of the global burden of rotavirus disease put the median cost of hospital stay in EU-25 Member States at €1,417 [9].

In Spain, two rotavirus vaccines are available (Rotateq^®^, Rotarix^®^), so far the Spanish public health authorities have not included them in the children Vaccination Recommendations. As there is no rotavirus-specific surveillance system in Spain, the data source used to estimate the frequency of hospital admissions was the Minimum Basic Data Set (MBDS), which is fully implemented in the Spanish health system [10]. Accordingly, from 1999 to 2000, winter incidence of hospital admissions due to rotavirus was 2.5 per 1000 children under 5 years of age, at an annual total cost of 3.6 million euros [10].

This study sought to describe and analyze hospital admissions in Spain due to rotavirus infections among children aged 5 years or under during the period 2001–2005, along with the associated total health cost.

## Methods

A retrospective, descriptive, epidemiologic study was conducted, using the MBDS as the data source. The MBDS is a national hospital admission database, managed by the Ministry of Health and showing all hospitalizations for which the diagnoses are coded according to the Spanish version of the International Classification of Diseases (ICD-9-CM) [11]. No ethical approval was required due to the fact that the data was publicly available. Aside from diagnoses at discharge (with the principal diagnosis shown first, and secondary diagnoses in the remaining positions), variables covered by the MBDS include: hospital identification; patients' identification, date of birth, gender, place of residence, and date of admission; surgical and obstetric procedures; other procedures; and, date and type of discharge. Estimated MBDS coverage is 95% of hospitals admissions nationwide [12,13].

We selected MBDS data corresponding to hospital admissions during the study period among children aged 5 years or under, with principal diagnosis at discharge of intestinal infectious gastroenteritis (ICD-9-CM codes 001 to 009 excluding 003.2, 006.3, 006.6) or noninfectious gastroenteritis (ICD-9-CM code 558.9). Based on ICD-9-CM codes 001–009, we then selected code 008.61, which specifically relates to rotavirus infection.

Incidence of hospitalization due to infectious and/or noninfectious gastroenteritis and rotavirus, and mean hospital stay were calculated, overall and stratified by age group. The population used as denominator for the calculation of incidence was obtained from a projection of the 2001 Spanish census for the study period, duly adjusted to the 95% of the population assigned to hospitals included in the MBDS [14]. Seasonal distribution of rotavirus admissions was compared using the Microbiologic Information System (MIS). The MIS is based on voluntary weekly reports of individual, microbiological case diagnoses performed by public-health-system laboratories. The coverage achieved by this system is low and geographically irregular, so data on the SIM cannot therefore be assumed to be representative of the population as a whole. However, these data are useful to describe trends, as the coverage did not change over the years [15]. We analyzed related comorbidity, mortality, and the direct medical cost to the health system of rotavirus hospitalizations. These costs were calculated using Diagnosis Related Groups (DRG) for this disease. According to the DRG reimbursement system, every hospitalized patient belongs to a group of diagnostically homogeneous cases; therefore patients within each category are similar clinically and are expected to use the same level of hospital resources. As a result patients in the same DRG group are assigned the same reimbursement charges [16].

A specific DRG for rotavirus gastroenteritis does not exist, therefore DRG 184 (esophagitis, gastroenteritis, and miscellaneous digestive disorders, age < 18) and 298 (nutritional and miscellaneous metabolic disorders, age < 18) were used (CMS-DRG version 22.0) [17].

All statistical analyses were performed using the SPSS statistics computer software package (version 14.0; Chicago, Illinois, USA). Quantitative variables were expressed as mean, median and standard deviation, and qualitative variables as frequency and percentage with 95% confidence intervals. Chi squared test was used to compare categorical variables and t tests to compare means. Statistical significance was set at p < 0.05 (p values are two-tailed).

## Results

A total of 95,054 children aged ≤ 5 years with infectious or noninfectious acute gastroenteritis (AGE) (789 cases per 100,000 population ≤ 5 years of age) were admitted to hospitals covered by the MBDS over the 5-year study period. Mean age of cases was 20.9 ± 17.4 months (median: 17 months), and 55.3% were males. There were 44 deaths among children hospitalized with AGE during the study period.

Of these hospitalizations, 54.7% were classified as infectious gastroenteritis (IAGE), and the remainder as noninfectious gastroenteritis. Shown in Table 1 is the etiology of admissions due to infectious gastroenteritis. In all, 38.1% of IAGE were of unspecified etiology; 34% of the remainder were accounted for by viruses (rotavirus: 31.2%), followed by bacteria (27.3%) and parasites (0.6%).

**Table 1:** Acute gastroenteritis by infectious etiology among children ≤ 5 years from year 2001 to 2005 according to Minimum Basic Data Set (MBDS).

	**Number of hospitalizations (%)**					
	
	**2001**	**2002**	**2003**	**2004**	**2005**	**2001–2005**
**Etiology unspecified**						
Presumed infectious	4,715 (42.9)	3,916 (41.7)	3,844 (38.2)	3,935 (36.7)	3,468 (31.7)	19,878 (38.1)
**Etiology specified**						
Viral total:	3,090 (28.1)	2,405 (25.5)	3,009 (29.8)	4,109 (38.3)	5,131 (46.8)	17,744 (34.0)
Rotavirus	2,977 (27.1)	2,129 (22.6)	2,723 (27.0)	3,718 (34.7)	4,708 (43.0)	16,255 (31.2)
Others	113 (1.0)	276 (2.9)	286 (2.8)	391 (3.6)	423 (3.8)	1,489 (2.8)
Bacterial total:	3,125 (28.4)	3,040 (32.3)	3,158 (31.3)	2,610 (24.3)	2,304(21.0)	14,237 (27.3)
*Salmonella*	1,942 (17.7)	1,892 (20.1)	2,074 (20.6)	1,743 (16.2)	1,479 (13.5)	9,130 (17.5)
Others	1,183 (10.7)	1,148 (12.2)	1,084 (10.7)	867 (8.1)	825 (7.5)	5,107 (9.8)
Parasitic	41 (0.6)	26 (0.5)	48 (0.7)	42 (0.7)	30 (0.5)	187 (0.6)

**Total**	**10,971**	**9,387**	**10,059**	**10,696**	**10,933**	**52,046**

Table 2 shows incidence, mean stay, and proportion of gastroenteritis due to rotavirus, by study year.

**Table 2:** Hospital admissions by acute gastroenteritis of any etiology and by rotavirus for years 2001–2005 according to Minimum Basic Data Set (MBDS).

	**Total admissions by acute gastroenteritis**			**Total admissions by rotavirus**			
**Year**	**Total**	**Mean Stay (SD)**	Incidence * 100,000 (CI95%)	**Total**	**Mean Stay (SD)**	Incidence * 100,000 (CI95%)	**% Acute gastroenteritis owed to rotavirus**

2001	19839	3.79 (3.08)	868 (856–880)	2977	4.30 (2.61)	130 (126–135)	15.0
2002	17882	3.73 (2.97)	776 (765–787)	2129	4.36 (3.39)	92(88–96)	11.9
2003	18565	3.86 (4.18)	776 (765–787)	2723	4.43 (4.51)	114(109–118)	14.6
2004	19527	3.73 (3.24)	784 (773–795)	3718	4.26 (3.08)	149(144–154)	19.0
2005	19241	3.68 (3.67)	745 (735–756)	4708	3.99 (2.43)	182(177–188)	24.4

**Total**	**95054**	**3.76 (3.45)**	**789 (784–794)**	**16255**	**4.23 (3.17)**	**135(133–137)**	**17.1**

SD: Standard deviation

CI: Confidence interval

A total of 17.1% of all admissions due to AGE of any etiology in children aged ≤ 5 years were attributable to rotavirus, with a total of 16,255 cases in the 5 years of study, indicating a mean incidence of 135 admissions per 100,000 children aged ≤ 5 years (95% confidence interval: 133–137). In 2005, this value rose to 24.4%, which meant 182 admissions per 100,000 children aged ≤ 5 years (95% confidence interval: 177–188). Mean hospital stay for rotavirus admissions was significantly higher than that for all-cause gastroenteritis admissions (4.2 ± 3.1 days vs. 3.7 ± 3.4 days). As from 2003, there was a significant reduction in mean stay (p ≤ 0.05, declining from 4.4 ± 4.5 days to 3.9 ± 2.4 days in 2005).

The mean age of children admitted for rotavirus was 14.5 ± 11.7 months (median:11 months). The proportion of hospital admissions due to rotavirus gastroenteritis among children in different age groups was examined, in order to ascertain age-specific rotavirus disease burden (Table 3). The lower the age, the higher the percentage of admissions due to rotavirus-related gastroenteritis (22% among infants under 1 year of age); by the same token, the percentage decreased among older children (3.3% at 5 years of age). Thus, incidence of hospital admissions among infants under 1 year age was superior to the rest of the age groups (Table 3). Infants under 1 year of age registered a mean hospital admission of 4.58 days (standard deviation: 3.8 days), which, as can be seen from Table 3, was significantly higher than that in any other age group.

**Table 3:** Total hospital admissions from 2001 to 2005 caused by acute gastroenteritis of any etiology and by rotavirus by age groups according to Minimum Basic Data Set (MBDS).

	**Total admissions by acute gastroenteritis**			**Total admissions by rotavirus**				
**Age group**	**Total**	**Mean stay (SD)**	Incidence * 100,000(CI95%)	**Total**	**Mean stay (SD)**	Incidence * 100,000 (IC95%)	**% Acute gastroenteritis owed to rotavirus**	**% by age group**

< 1 year	35819	4.16 (4.10)	1682(1664–1699)	8188	4.58 (3.88)	384 (376–393)	22.8	50.4
1 year	27385	3.60 (3.44)	1318(1303–1334)	5437	3.98 (2.33)	262 (255–269)	19.8	33.4
2 years	13786	3.49 (2.58)	682 (671–694)	1674	3.68 (1.85)	83 (79–87)	12.1	10.3
3 years	7759	3.48 (2.53)	393 (383–401)	530	3.78 (1.87)	27 (24–29)	6.8	3.3
4 years	5738	3.34 (2.29)	296 (288–303)	273	3.40 (1.70)	14 (12–16)	4.7	1.7
5 years	4567	3.34 (2.47)	239 (232–246)	153	3.40 (1.51)	8 (7–9)	3.3	0.9

SD: Standard deviation

CI: Confidence interval

**Figure 1:**
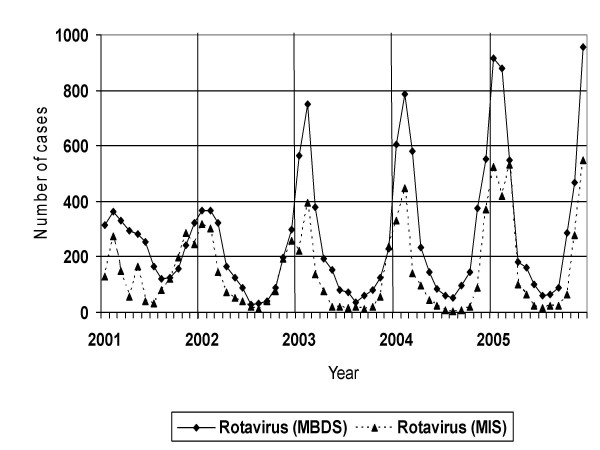
**Laboratory identifications rotavirus reports compared with hospital admissions attributable to rotavirus (2001–2005)**. Distribution from year 2001 to 2005 of the laboratory identifications rotavirus reports according to the Microbiologic Information System (MIS) compared with hospital admissions attributable to rotavirus in children ≤ 5 years of age according to Minimum Basic Data Set (MBDS).

Temporal distribution of rotavirus infections across the study period, by month of admission (Figure 1), displayed a clear predominance in winter months (December, January and February). Number of hospitalizations during the winter months was superior than in summer months (June, July, August) (8,268 cases vs. 1,307 in children aged ≤ 5 years for the 2001–2005 period). Furthermore, data furnished by the MIS show the same seasonal distribution.

A total of 36.4% of children admitted with principal diagnosis of rotavirus had secondary diagnoses: and of these, 52% presented with weight loss, 13.7% with acidosis, and the remaining 34% with other diagnoses.

Of the 16,255 children admitted for rotavirus-related intestinal infection during the 5 years of the study, 8 died as a consequence of this infection (0.04%).

**Figure 2:**
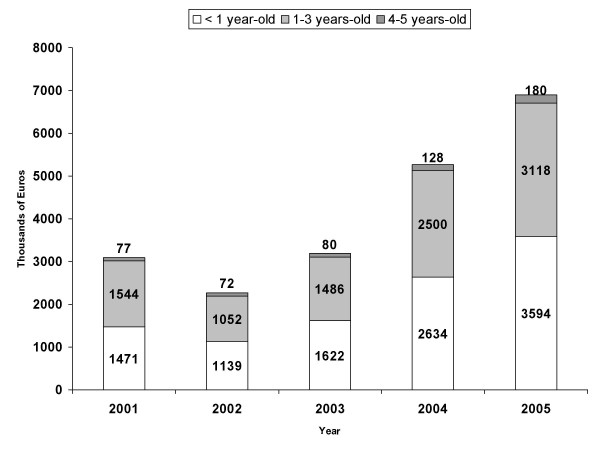
**Total cost trend for rotavirus-related admissions (2001–2005)**. Total cost trend for rotavirus-related admissions in children ≤ 5 years-old by age group, from year 2001 to 2005.

Using DRG for this disease, the estimated cost of each rotavirus-related hospital admission per year was €1,039.21 in 2001, €1,063.54 in 2002, €1,171.34 in 2003, €1,415.87 in 2004, and €1,464.14 in 2005. Shown in Figure 2 is the total cost trend for all rotavirus-related admissions over the 5-year study period, by age group. Over the entire study period, total hospital costs were higher for infants under 1 year of age than for the remaining age groups. In this age group, the total cost of hospital admission in 2005 was more than twofold that of 2001 (€3,594,000 vs. €1,471,000).

## Discussion

Across the 5 years of our study, incidence of rotavirus-related hospital admissions in Spain among children aged ≤ 5 years was 135 per 100,000, higher than that described for the period 1999–2000, which put it at 103.9 per 100,000 [10]. In our study, incidence rose in 2005 to 182 per 100,000 children aged ≤ 5 years. Broken down by age group, this value was 384 per 100,000 among infants under 1 year of age, and 262 per 100,000 among children aged 1–2 years. These data are lower than the European mean (300 per 100,000 children aged under 5 years, range: 30–1190 per 100,000), described in the Pediatric Rotavirus European Committee's 2006 review [9]. This difference can be attributed to two factors. First, hospital admission and health-system access policies differ among the various European countries. Second, results may be influenced by the design of the study undertaken, on including data obtained on the basis of direct or indirect estimates using microbiologic and hospital record data, as well as data from prospective studies.

Our study data show that in Spain, among all children aged ≤ 5 years with AGE as principal diagnosis, the proportion of hospital admissions due to rotavirus has risen, i.e., from 13.5% in the period 1999–2000 [10] to 17.1% in the study period (2001–2005). This last finding is in line with a study conducted in the USA from 1993–2002, using a methodology similar to ours, in which a mean value of 18% associated with rotavirus infection was reported [4]. Furthermore, in Europe, 10.4%–36.0% of all children aged ≤ 5 years admitted with AGE in the period 2004–2005 were hospitalized as a consequence of rotavirus [18]. Though we have defined a case of rotavirus by a specific ICD code, 008.61, and not by microbiological diagnosis, we considered that this code was likely given only for cases where rotavirus was detected in the stool of the patient. We consider that one reason for the increasing trend in rotavirus may be that physicians are sending more stool of the patient to be laboratory for a microbiological analysis.

Of all the infectious types of gastroenteritis that we studied, approximately one third of admissions corresponded to rotavirus, a figure similar to that described in different American and European studies [3,19-21].

In our study, as in the case of the USA and Europe, risk of hospitalization due to rotavirus was higher among children under two years of age [4,9]. Mean hospital stay among children under 5 years of age, albeit slightly shorter than the European mean (4.8 days), lies within the European range (2–9.5 days) [9].

As in other European countries and the USA, during the annual epidemic period rotavirus causes an excess of hospitalizations due to gastroenteritis [4,22-24]. Indeed, in Spain, 13.9% of all positive cultures reported to the MIS during the study period corresponded to rotavirus, there being a clear seasonal distribution in evidence, with peak incidence in winter, liked in other studies with different methodology [8,25]

Soriano-Gabarro and coworkers [6] estimated that 7 rotavirus deaths occur annually in Spain, using a model developed by the US Centers for Disease Control and Prevention [1]. This value is higher than that obtained in our study, inasmuch as 8 deaths were reported over a period of 5 years. This could be explained by the fact that their estimates, rather than being solely based on Spanish data, were arrived at on the basis of per capita gross national income [6].

The cost of hospital admission in Spain for children under 5 years of age has risen from the 3.6 million euros estimated for the period 1999–2000 [10] to almost 7 million euros estimated in our study for 2005. Although the greater part of rotavirus diseases are successfully treated in a primary care setting in Spain, hospitalization-related costs nevertheless account for approximately 80% of total medical costs associated with rotavirus infection [8].

This study has a number of limitations. In the first place, a national administrative database was used as the data source, since in Spain rotavirus infections are not subject to special surveillance, nor are they statutorily notifiable, which means that information may not be collected from all Spanish hospitals or that the information may be incomplete. Furthermore as data from the MBDS is anonymous it is impossible to identify if the same child is hospitalized more than once in the same year. However, the MBDS, which was first introduced in 1982, is a mandatory register and has an estimated coverage of as high as 95% [11]. Secondly, only rotavirus cases whose code appeared in the first diagnosis were selected from the database, with the result that the number of cases might be underestimated. The reason given be other authors to explain this underestimation include: paediatricians in many hospitals may have been discouraged from ordering diagnostic tests for rotavirus that increase the cost of medical care but not significantly alter treatment decisions; therefore it is possible that not all the patients undergo a stool analysis; and that some laboratories do not have sensitive enough tests for the detection of the virus [3,26,27]. We nevertheless consider that the use of this database was justified, on its having been developed as a useful instrument for ascertaining the mean stay of patients admitted to Spanish hospitals [28] as in other countries of our environment [26,27]. Thirdly, both the incidence and proportion of acute gastroenteritis represented by rotavirus may possibly have risen as a consequence of health professionals showing heightened interest in this microorganism in recent years and, by extension, requesting microbiologic confirmation in a greater proportion of AGE cases.

## Conclusion

In conclusion, this study provides updated information on the extent of diseases and the cost of rotavirus-specific hospitalizations in Spain. Our results indicate that gastroenteritis caused by rotavirus in Spain is an important health problem, particularly among children aged under 2 years, mostly during the winter season.

## Competing interests

The author(s) declare that they have no competing interests.

## Authors' contributions

ALA has participated in conceive the study, has made substantial contribution to analysis and interpretation of the data, and has been involving in writing the manuscript. RJG has been involving in writing the manuscript and revising it critically for important intellectual content. PCG has been involving in writing the manuscript and revising it critically for important intellectual content. AAM has made substantial contribution to analysis the data. PGG has made substantial contribution to analysis the data. AGM has conceived the study, has been involving in writing and participated in its design and coordination and helped to draft the manuscript and revising it critically for important intellectual content.

All authors read and approved the final manuscript.

## Pre-publication history

The pre-publication history for this paper can be accessed here:


